# Changes in Screening Test Volume in the National Breast and Cervical Cancer Early Detection Program during the COVID-19 Pandemic, 2020–2022

**DOI:** 10.3390/ijerph21070816

**Published:** 2024-06-21

**Authors:** Yamisha Bermudez, Amy DeGroff, Jacqueline Miller, Kristy Kenney, Jala Lockhart, Djenaba Joseph, Lisa Richardson

**Affiliations:** 1 Totally Joined for Achieving Collaborative Techniques, Atlanta, GA 30303, USA; 2 Division of Cancer Prevention and Control, National Center for Chronic Disease Prevention and Health Promotion, Centers for Disease Control and Prevention, Atlanta, GA 30341, USA

**Keywords:** breast cancer, cervical cancer, cancer screening, cancer screening program, COVID-19

## Abstract

Introduction: The National Breast and Cervical Cancer Early Detection Program (NBCCEDP) observed significant declines in screening volume early in the COVID-19 pandemic, January–June 2020, with variation by race/ethnicity and geography. We aimed to determine how screening in the NBCCEDP recovered from these early declines as it is important for monitoring the long-term impact on women served by the program. Methods: Extending the previous analyses, we compared monthly breast (BC) and cervical cancer (CVC) screening volume in the NBCCEDP during 2020–2022, to five-year, pre-COVID-19 pandemic averages (2015–2019), and calculated percent change. Results were stratified by race/ethnicity and rurality groups. We employed multiple one-way ANOVA tests, which included multiple comparisons, to test for significant differences between groups. Results: By December 2022, NBCCEDP breast and cervical cancer screening volumes had not fully recovered to pre-COVID-19 5-year averages, and recovery in breast cancer screening volume was slower than that of cervical cancer. Both BC and CVC screening among women in metro areas showed the smallest average monthly deficits (−8.8% BC and −4.9% CVC) compared to monthly pre-COVID-19 pandemic 5-year averages, and screening among women in rural areas showed the greatest deficits (−37.3% BC and −26.7% CVC). BC and CVC screening among Hispanic women showed the greatest improvements compared to the pre-COVID-19 averages (8.2% BC and 9.5% CVC), and cervical cancer screening among non-Hispanic Asian and Pacific Islander women showed the greatest deficits (−41.4% CVC). Conclusion: For increased intervention efforts, NBCCEDP recipients can focus on populations demonstrating greatest deficits in screening volume.

## 1. Introduction

In the United States, while millions of Americans were sickened or died from COVID-19 [1,2,3], delivery of primary health care services was also negatively impacted [4,5], including breast and cervical cancer screening. Screening for breast and cervical cancer has been shown to reduce mortality and is recommended by the United States Preventive Services Task Force [6,7]. During January–April 2020, there was an approximately 94% decrease in both breast and cervical cancer screenings in the United States [8]. Recent predictive modeling studies suggest that delayed or missed cancer screening due to the COVID-19 pandemic can result in increased late-stage breast cancer incidence [9,10] and mortality [11,12,13]. Further, existing cancer-related disparities among women who identify as racial and ethnic minorities and others who are medically underserved [14,15] could be exacerbated by the COVID-19 pandemic given that these groups have disproportionately higher COVID-19 incidence rates [16].

The National Breast and Cervical Cancer Early Detection Program (NBCCEDP), administered by the Centers for Disease Control and Prevention, provides breast and cervical cancer screening and diagnostic services to women with low incomes who are largely uninsured. Specifically, women meet NBCCEDP eligibility requirements if they are uninsured, aged 40–64 years for breast screening, aged 21–64 years for cervical screening, and live in households with incomes at or below their state-established federal poverty limit (FPL). Since its inception in 1991, the NBCCEDP has served more than 6.2 million program-eligible women and provided more than 16.1 million breast and cervical cancer examinations [17]. The NBCCEDP is carried out by award recipients in all 50 states, the District of Columbia, 13 tribes or tribal organizations, 2 US territories, and 5 US-Affiliated Pacific Islands. Nearly three-quarters of women served through the program belong to racial and ethnic minority groups and all are low-income and mostly uninsured.

A CDC-led study examining the impact of the COVID-19 pandemic on NBCCEDP screening volume during January–June 2020 observed declines of over 80% in both breast and cervical cancer screening tests in April 2020 compared to the previous 5-year average for that month [18]. The study found the greatest declines in screening volume among non-Hispanic American Indian/Alaskan Native (AI/AN) women for breast cancer screening and non-Hispanic Asian and Pacific Islander (AAPI) women for cervical cancer screening. When assessing geographic differences in screening volume, the authors also found that declines were greatest in rural areas and in Health and Human Services Region 2—New York. A later geographic assessment of changes in NBCCEDP screening volume found that, among states that consistently experienced high COVID-19 test percent positivity during a 6-month period (July–December 2020), six concurrently experienced large decreases in both breast and cervical cancer screening volume [19].

The long-term impact of the COVID-19 pandemic on NBCCEDP screening volume is unknown. The goal of this study was to further examine the impact of the COVID-19 pandemic on the NBCCEDP by assessing whether deficits in screening volume persisted or increased. Specifically, this paper extends the previous screening volume analyses through 2022, providing a full three years of data (January 2020–December 2022). The following research questions were addressed:

1. How do monthly breast and cervical cancer screening volume in 2020, 2021, and 2022 compare to pre-COVID-19 pandemic, 5-year monthly averages (2015–2019)?
2. Do the monthly comparisons differ by race/ethnicity and rurality?

## 2. Materials and Methods

For this descriptive study, we used NBCCEDP program data (minimum data elements (MDEs)) that NBCCEDP recipients submit to CDC on all women receiving services through the NBCCEDP. Each MDE record describes patient demographics; screening test type performed and date; results; final diagnosis; and date of cancer treatment initiation, if indicated [20]. The MDE data collection was reviewed and approved by the CDC Institutional Review Board. The MDE data collection was also approved by the Office of Management and Budget (OMB, #0920-1046, exp. 03.31.2025).

We determined the number of breast screening tests (mammography) and cervical screening tests (Papanicolaou [Pap] tests, human papillomavirus [HPV] testing, or HPV/Pap co-testing) delivered monthly for each year starting with 2015 through 2022. A five-year monthly average was calculated for years (2015–2019) to represent the same pre-COVID-19 pandemic period used in the previous analysis examining changes in screening volume during the first 6 months of the COVID-19 pandemic [18]. We used 2015–2019 as the pre-COVID-19 comparison period to be consistent with the previous analysis and because it represents screening volume data which has not been impacted by the COVID-19 pandemic. Using the average of a full 5 years of data as the baseline comparison provides the most adequate representation of screening volume in the program prior to the COVID-19 pandemic. Therefore, we compared five-year monthly averages to monthly screening volume for 2020, 2021, and 2022. NBCCEDP recipients in North Carolina, Marshall Islands, American Indian Cancer Foundation, Great Plains, and Cheyenne River Sioux were excluded because 2020 data were not submitted. Data were then stratified based on race/ethnicity and rurality. We combined race and ethnicity to create seven mutually exclusive race/ethnicity categories: non-Hispanic White, non-Hispanic Black, non-Hispanic AAPI, non-Hispanic AI/AN, multiracial, Hispanic, and unknown. We used the U.S. Department of Agriculture Urban Rural Continuum Codes to assign each MDE record to one of three categories: metropolitan (metro), urban, or rural. We determined the percentage change between monthly screening volume compared to pre-COVID-19 pandemic, 5-year monthly averages. For this study, negative percentage change values represent deficits in monthly screening volume compared to pre-COVID-19 pandemic, 5-year monthly averages. Positive percentage change values represent improvements in monthly screening volume compared to pre-COVID-19 pandemic, 5-year monthly averages.

Using SAS 9.4, we carried out two separate one-way ANOVA tests, which included the Student–Newman–Keuls multiple-range test for multiple comparisons, to determine if there were significant differences in the average monthly percent change in breast cancer screening volume compared to pre-COVID-19 pandemic, 5-year monthly averages across rurality (test 1) and racial/ethnic groups (test 2) during January 2020–December 2022 [21]. The same tests were employed for cervical cancer screening volume.

## 3. Results

**Figure 1:**
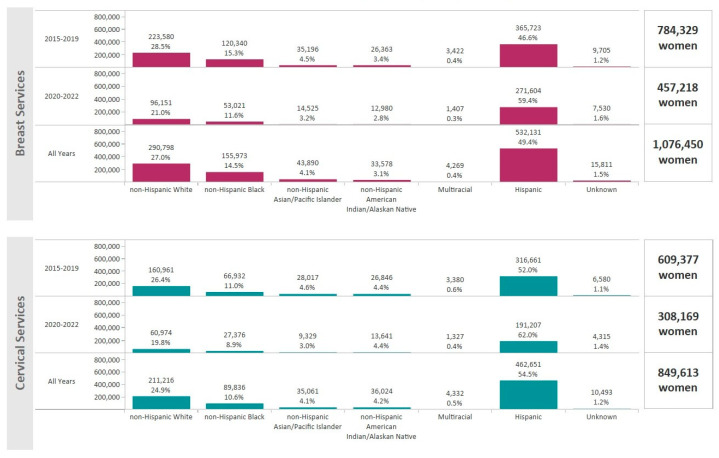
Total number of women that received breast and cervical cancer screenings through the NBCCEDP during 2015–2022 by race/ethnicity. The dark pink bars represent breast cancer screening volume and the light blue bars represent cervical cancer screening volume.

**Figure 2:**
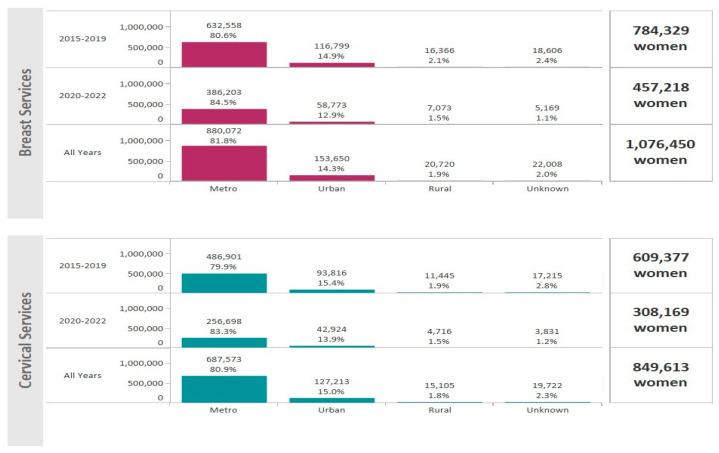
Total number of women that received breast and cervical cancer screenings through the NBCCEDP during 2015–2022 by rurality. The dark pink bars represent breast cancer screening volume and the light blue bars represent cervical cancer screening volume.

For the period January 2015–December 2022, the NBCCEDP provided 1,721,804 mammograms and 1,632,693 Pap tests and/or HPV tests. A total of 1,076,450 women received breast cancer screening and 849,613 women received cervical cancer screening services (Figure 1). Hispanic women comprised 49.4% of breast cancer screenings and 54.5% of cervical cancer screenings (Figure 1). The majority of women receiving breast and cervical cancer screening lived in metro areas (81.8% and 80.9%, respectively) (Figure 2).

**Figure 3:**
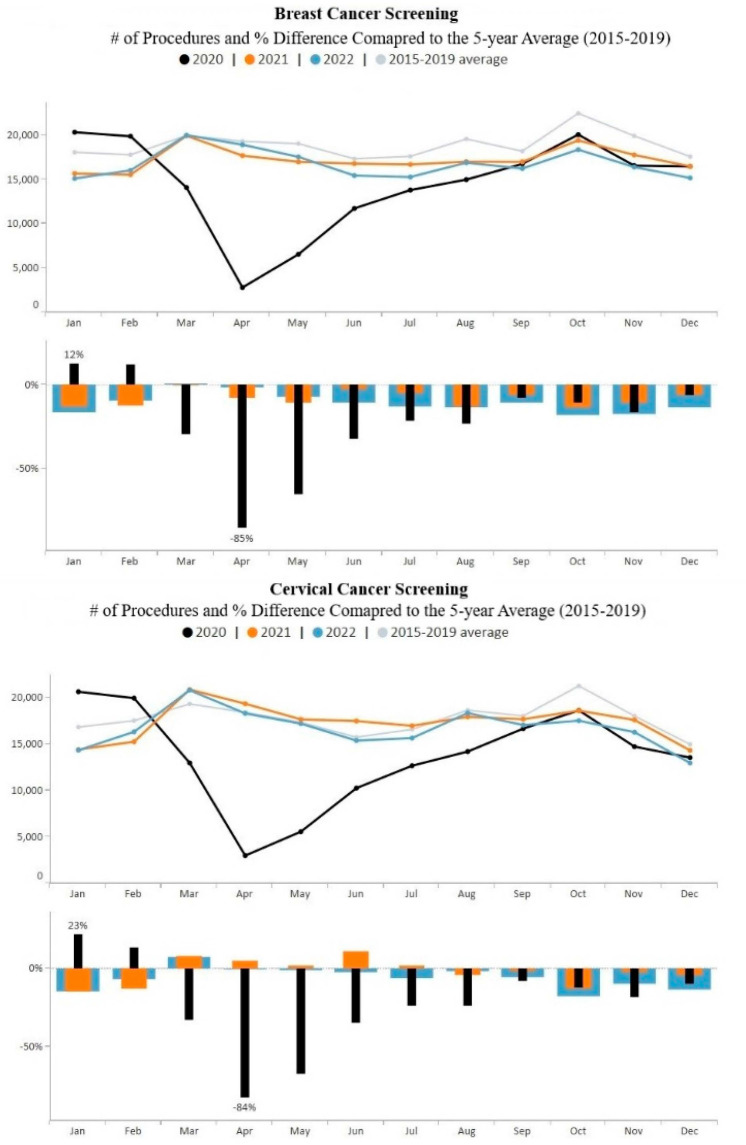
Monthly NBCCEDP breast and cervical cancer screening tests for January 2020 through December 2022 compared with the 5-year average, 2015–2019. The grey line represents average screening tests conducted over the 5-year period, 2015–2019; black line represents screening tests conducted in 2020; orange line represents screening tests conducted in 2021; blue line represents screening tests conducted in 2022. The same color representation applies to histograms. Histograms above the 0% line represent monthly screening volumes that have increased by various percentage points compared to the 5-year averages (2015–2019); those below the 0% line represent monthly screening volumes that have decreased by various percentage points compared to the 5-year averages (2015–2019).

Figure 3 provides overall monthly breast and cervical cancer screening volume data for the study period. Screening volume dropped precipitously in April 2020 for both breast and cervical cancer. Trends for both breast and cervical cancer show increasing volume from April to October 2020 and remained relatively stable throughout 2021. However, from June to December 2022, monthly breast and cervical cancer screening volumes fell below 2021 monthly screening volumes, as well as below the pre-COVID-19 averages.

Figure 3 also shows that overall cervical cancer screening volume increased closer to pre-COVID-19 pandemic 5-year averages than breast cancer screening volume. During the five-month period, March–July 2021, cervical cancer screening volume exceeded the 5-year average. During 2021 and 2022, monthly cervical cancer screening volume was an average of 2% and 6% below pre-COVID levels, respectively while breast cancer screening volume was an average of 8% and 11% below pre-COVID averages, respectively. Both breast and cervical cancer screening volume remained below pre-COVID-19 pandemic averages during the last five months of 2022.

**Figure 4:**
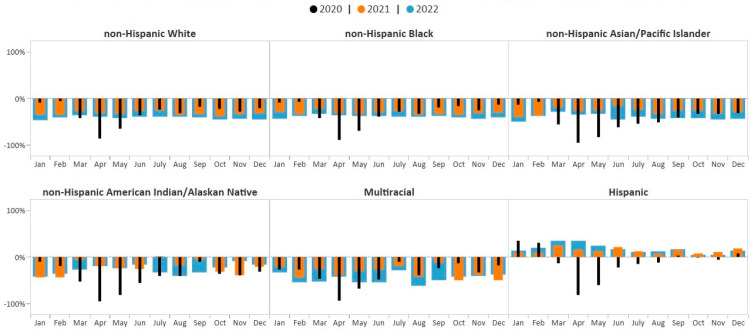
Monthly NBCCEDP breast cancer screening tests for January 2020 through December 2022 compared with the 5-year average, 2015–2019, by race/ethnicity. The grey line represents average screening tests conducted over the 5-year period, 2015–2019. Black histograms represent screening tests conducted in 2020; orange histograms represent screening tests conducted in 2021; blue histograms represent screening tests conducted in 2022. Histograms above the 0% line represent monthly screening volumes that have increased by various percentage points compared to the 5-year averages (2015–2019); those below the 0% line represent monthly screening volumes that have decreased by various percentage points compared to the 5-year averages (2015–2019).

One-way ANOVA results revealed that there was a statistically significant difference in average monthly percent change in breast cancer screening volume between at least two racial/ethnic groups (F (5, 186) = 51.95, *p* < 0.0001) during the study period, January 2020–December 2022. The Student–Newman–Keuls multiple-range test for multiple comparisons showed that the average monthly percent change in breast cancer screening volume was significantly different among Hispanic women compared with women in all other racial/ethnic groups (*p* < 0.05). The average monthly breast cancer screening volume among Hispanic women showed the greatest improvements compared to pre-COVID-19 averages (8.2%) during the study period and showed sustained improvement over pre-COVID-19 pandemic averages beginning in December 2020 (7% above pre-COVID-19 pandemic average) through December 2022 (13% above pre-COVID-19 pandemic average) (Figure 4). In contrast, breast cancer screening volume for all other racial/ethnic groups remained consistently below monthly pre-COVID-19 pandemic averages throughout the study period, 2020–2022.

**Figure 5:**
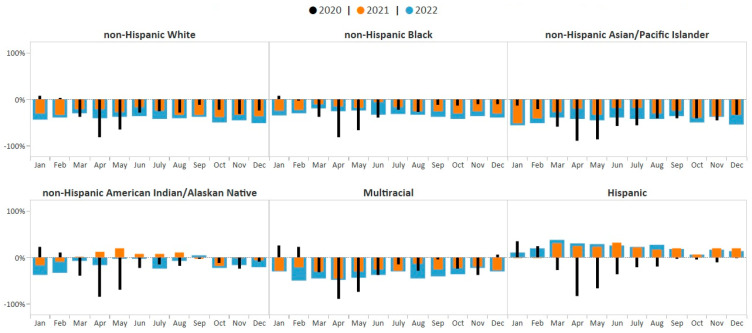
Monthly NBCCEDP cervical cancer screening tests for January 2020 through December 2022 compared with the 5-year average in 2015–2019, by race and ethnicity. The grey line represents average screening tests conducted over the 5-year period, 2015–2019. Black histograms represent screening tests conducted in 2020; orange histograms represent screening tests conducted in 2021; blue histograms represent screening tests conducted in 2022. Histograms above the 0% line represent monthly screening volumes that have increased by various percentage points compared to the 5-year averages (2015–2019); those below the 0% line represent monthly screening volumes that have decreased by various percentage points compared to the 5-year averages (2015–2019).

There was also a statistically significant difference in the average monthly percent change in cervical cancer screening volume between racial/ethnic groups (F (5, 185) = 45.24, *p* < 0.0001) during the study period. The Student–Newman–Keuls test found that the average monthly percent change in cervical cancer screening volume was significantly different among Hispanic, non-Hispanic AI/AN, and non-Hispanic AAPI women compared with women in all other racial/ethnic groups (*p* < 0.05). The average monthly cervical cancer screening volume among Hispanic women showed the greatest improvements compared to pre-COVID-19 averages (9.5%, *p* < 0.05), with screening volume above pre-COVID-19 pandemic averages during all months of 2021 and 2022 (Figure 5). The average monthly cervical cancer screening volume among non-Hispanic AI/AN women showed the smallest deficit (−11.6%, *p* < 0.05). Cervical cancer screening among non-Hispanic AI/AN women rose to 20% above the pre-COVID-19 pandemic average in May 2021; however, by September 2021, the screening volume fell back below the pre-COVID-19 pandemic average and remained below the baseline through December 2022 (Figure 5). Cervical cancer screening volume for all other racial/ethnic groups remained below monthly pre-COVID-19 pandemic averages during all of 2021–2022 (Figure 5). Average monthly percent change in cervical cancer screening volume was not significantly different among women who identified as non-Hispanic Black, non-Hispanic White, and Multiracial compared with women in all other racial/ethnic groups. However, the average monthly percent change in screening volume among non-Hispanic AAPI women showed the greatest deficit (−41.4%, *p* < 0.05).

**Figure 6:**
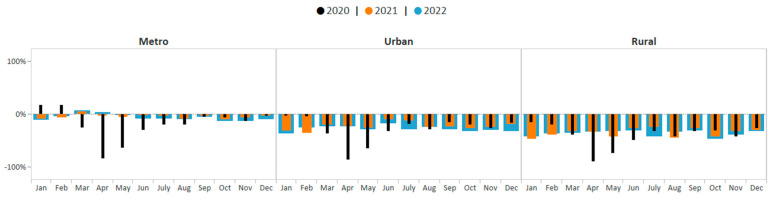
Monthly NBCCEDP breast cancer screening tests for January 2020 through December 2022 compared with the 5-year average in 2015–2019, by rurality. The grey line represents average screening tests conducted over the 5-year period, 2015–2019. Black histograms represent screening tests conducted in 2020; orange histograms represent screening tests conducted in 2021; blue histograms represent screening tests conducted in 2022. Histograms above the 0% line represent monthly screening volumes that have increased by various percentage points compared to the 5-year averages (2015–2019); those below the 0% line represent monthly screening volumes that have decreased by various percentage points compared to the 5-year averages (2015–2019).

One-way ANOVA test results suggest that the average monthly percent change in breast cancer screening volume was statically significantly different across rurality groups during January 2020–December 2022 (F (2, 93) = 57.64; *p* < 0.0001). The Student–Newman–Keuls test suggests that average monthly screening volume among metro, rural, and urban areas were all significantly different from each other during this period (*p* < 0.05). The average monthly breast cancer screening volume among women in metro areas showed the smallest deficit (−8.8%, *p* < 0.05). Breast cancer screening volume in metro areas improved from 85% below the five-year average in April 2020 to only 2% below the five-year average by December 2021 (Figure 6). Consistent with the aggregate data, breast cancer screening volume among women in metro areas then declined slightly in 2022 yet remained closer to pre-COVID-19 pandemic averages compared to screening volume in urban and rural areas. The average monthly breast cancer screening volume among women in urban areas showed the second greatest deficit (−26.1%), *p*-value < 0.05. Breast cancer screening volume among women living in urban areas remained below pre-COVID-19 pandemic averages during each month of 2021–2022; however, monthly deficits were not as great as those observed among women living in rural areas. The average monthly breast cancer screening volume among women in rural areas showed the greatest deficit (−37.3%, *p* < 0.05). Breast cancer screening volume among women living in rural areas also remained below pre-COVID-19 pandemic averages during each month of 2021–2022. By December 2022, breast cancer screening volume was below pre-COVID-19 pandemic averages by 33%.

**Figure 7:**
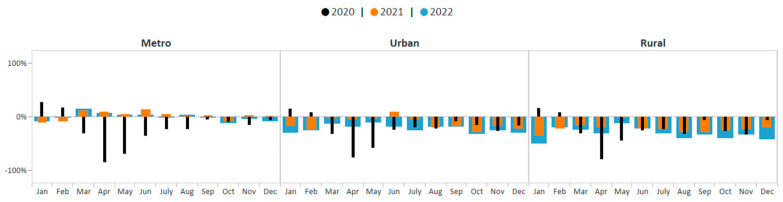
Monthly NBCCEDP cervical cancer screening tests for January 2020 through December 2022 compared with the 5-year average in 2015–2019, by rurality. The grey line represents average screening tests conducted over the 5-year period, 2015–2019. Black histograms represent screening tests conducted in 2020; orange histograms represent screening tests conducted in 2021; blue histograms represent screening tests conducted in 2022. Histograms above the 0% line represent monthly screening volumes that have increased by various percentage points compared to the 5-year averages (2015–2019); those below the 0% line represent monthly screening volumes that have decreased by various percentage points compared to the 5-year averages (2015–2019).

Similar patterns were observed when assessing changes in cervical cancer screening volume by rurality groups. One-way ANOVA test results indicate that the average monthly percent change in cervical cancer screening volume was not the same across rurality groups during the study period (F (2, 93) = 22.36; *p* < 0.0001). Average monthly cervical cancer screening volume among metro, rural, and urban areas were all significantly different from each other during this period (*p* < 0.05). The average monthly cervical cancer screening volume among women in metro areas showed the smallest deficit (−4.9%, *p* < 0.05). Cervical cancer screening volume among women in metro areas improved from 85% below the five-year average in April 2020 to 1% above the 5-year average by December 2021 (Figure 7). Consistent with the aggregate data, cervical cancer screening volume among women in metro areas then declined slightly in 2022 yet remained closer to pre-COVID-19 pandemic averages compared to screening volume in urban and rural areas. The average monthly cervical cancer screening volume among women in urban areas showed the second greatest deficit (−19.3%, *p* < 0.05). Except for in June 2021, cervical cancer screening volume among women living in urban areas also remained below pre-COVID-19 pandemic averages during all of 2021–2022; however, monthly deficits were not as great as those observed among women living in rural areas. The average monthly cervical cancer screening volume among women in rural areas showed the greatest deficit (−26.7%, *p* < 0.05). Cervical cancer screening volume among women living in rural areas remained below pre-COVID-19 pandemic averages during each month of 2021–2022. By December 2022, the screening volume was 42% below pre-COVID-19 pandemic averages for cervical cancer.

## 4. Discussion

Recovering cancer screening volume during the COVID-19 pandemic has been an ongoing challenge; especially among lower-income populations [22]. Three years after the COVID-19 pandemic began, breast and cervical cancer screening volumes among NBCCEDP’s unique population of women with low incomes who are largely uninsured, and identify as a racial and ethnic minority, had not fully recovered to pre-COVID-19 pandemic, 5-year averages. At the end of the study period, December 2022, overall breast and cervical cancer screening volumes remained 14% and 13% below the pre-COVID-19 pandemic 5-year averages for that month, respectively. Screening recovery also differed by service type, race/ethnicity, and rurality. Understanding the long-term impacts of the COVID-19 pandemic on the program’s screening volume is important as it may inform strategies that can help to reduce the risk of this population experiencing exacerbated cancer outcomes.

Over the 3-year study period, we found that monthly cervical cancer screening volume increased closer to pre-COVID-19 pandemic, 5-year averages compared to monthly breast cancer screening volume. During 2021 and 2022, monthly cervical cancer screening volume was an average of 2% and 6% below pre-COVID-19 pandemic levels, respectively. In contrast, breast cancer screening volume was an average of 8% and 11% below pre-COVID averages, respectively. Two factors may partially explain this discrepancy. First, in early 2021, the Society of Breast Imaging (SBI) recommended postponing breast imaging until at least four to six weeks following completion of the COVID-19 vaccine schedule to prevent unnecessary workups and biopsies given radiologists observed a higher incidence of axillary lymphadenopathy on screening mammography images [23,24] Although the SBI recommendation was revised in February 2022, it may have contributed to the deficit in breast cancer screening volume. A second factor that may explain this discrepancy involves imaging facilities limiting the number of mammograms offered and prioritizing women at higher risk for breast cancer during the pandemic, resulting in a significant backlog in appointments for screening mammograms [25]. In contrast, appointment backlogs may be less relevant for Pap testing as these tests can be provided in primary care offices, which significantly outnumber imaging facilities.

Another observed pattern from the study involves declines in both breast and cervical cancer screening volumes in the second half of 2022. Following the dramatic decline in March and April 2020, both breast and cervical cancer screening volumes steadily increased from April to October 2020 and remained relatively stable throughout 2021. However, from June to December 2022, monthly volumes fell below 2021 monthly screening volumes, as well as below the pre-COVID-19 pandemic averages. These declines may be a consequence of increased COVID-19 community levels in the US during 2022 [26,27,28] and related fear of COVID-19 exposure [29,30]. The maximum COVID-19 test percent positivity in 2020 was 15.7%, and in 2021 it was 14.4% [27]. However, the maximum COVID-19 test percent positivity in 2022 was 30.2% [27]. Although there are currently no data describing the relationship between yearly changes in COVID-19 community levels and fear of COVID-19 during 2020–2022, early findings suggest that patient fear of contracting COVID-19 negatively impacted providers’ ability to perform cancer screenings for breast and cervical cancer [29,30].

Declines in the NBCCEDP screening volume in 2022 may also be related to reduced capacity in the health system due to COVID-19 pandemic-related burnout and resignations among health care providers [31,32]. Data from the Health Care Worker Stress Survey administered in late 2020 indicate that, among 1119 healthcare workers, 76% felt exhaustion and burned out, and 55% questioned their career path due to the COVID-19 pandemic [31]. A recent report indicates that by November 2022, healthcare and social assistance resignation rates were 32.5% higher compared to pre-COVID-19 pandemic rates [32]. This pandemic-related loss of healthcare workers likely exacerbated a pre-existing provider shortage [33], which may have contributed to the observed declines. States enacted the 1135 Medicaid waiver in response to the COVID-19 pandemic, which temporarily waived or modified Medicaid eligibility, pre-approval, and certification requirements during 2020–2023 [34]. The waiver extended Medicaid coverage for many women and ultimately reduced the number of women that were uninsured and thus eligible for the NBCCEDP.

NBCCEDP breast and cervical cancer screening volumes in rural areas did not recover as well as in urban and metro areas. Provider shortages in rural communities existed well before the COVID-19 pandemic [35]. However, with providers experiencing high turnover and burnout due to the COVID-19 pandemic, in addition to rural hospitals closing [31,32,36], the shortage of providers in rural areas has been exacerbated [37,38]. Between 2021 and 2022, the percentage of primary care health professional shortage areas located in rural areas increased from 61% to 65.6% [37,38]. Further, the large number of rural hospital closures during the COVID-19 pandemic, and subsequent increased accessibility challenges for patients (i.e., traveling greater distances for care), may have also negatively impacted screening in rural areas [36,39].

In this study, cervical cancer screening volume among women who are non-Hispanic Asian and Pacific Islander did not recover as well to pre-COVID-19 averages compared to all other racial/ethnic groups. In contrast, both breast and cervical screening volumes among Hispanic women exceeded the 5-year pre-COVID monthly averages throughout all of 2021 and 2022. A 2021 study indicated that the mean score for fear of COVID-19 on an internet panel survey was 2.71 among Hispanics compared to 3.00 and 3.26 among African Americans and Whites, respectively, with lower scores indicating less fear [40]. Lower levels of fear of COVID-19 among Hispanics may partially explain why the screening volume among Hispanic women exceeded the 5-year pre-COVID monthly averages in 2021–2022. However, the literature on this topic is mixed, with other studies finding that Hispanics had a higher perceived threat of COVID-19 [41,42].

A recent case study found that among the six NBCCEDP recipients that were able to maintain breast and/or cervical cancer screening during periods of high COIVD-19 test positivity (Arkansas Breast Care, Iowa Care for Yourself, Kansas Early Detection Works, Nevada Women’s Health Connection, South Carolina Best Chance Network, and Tennessee Breast and Cervical Screening Program), there were a number of strategies implemented that may have aided in their success [43]. Key strategies include (1) modified protocols to address patient safety and fear of COVID-19, (2) enhanced patient reminders, (3) increased education regarding the importance of staying up-to-date with cancer screening during the COVID-19 pandemic, and (4) increased efforts to identify and reduce patients’ structural barriers [43]. These are strategies that may be helpful to other NBCCEDP recipients as they attempt to improve screening volume among the identified populations of women experiencing the greatest deficits in screening volume compared to pre-COVID-19 averages. It may be especially important for NBCCEDP recipients to increase efforts to identify and reduce patients’ barriers, as unique barriers may be contributing to the observed racial/ethnic and geographic differences. Additionally, implementing tailored outreach and support for women who are racial/ethnic minorities may be necessary to engage women in screening. Patient navigation may also be helpful in facilitating women’s access to mammography, which could help to improve breast cancer screening volume, especially as screening backlogs are resolved. Lastly, recipients could address screening in rural areas by assisting patients with transportation, especially those having to travel a significant distance to a healthcare facility. It may also be important to address the broader issue of provider shortage in rural areas, and HRSA’s programs to support and grow the healthcare workforce in rural communities has the potential to make a significant impact [44,45].

There are some study limitations. First, there were small numbers of women enrolled within certain racial/ethnic groups; therefore, we had to conduct our analysis using some combined categories. Secondly, deficits in breast and cervical cancer screening volumes during the study period cannot be solely attributed to the COVID-19 pandemic. Other contributing factors that were not explored in this study may be associated with the observed changes in screening volume. For example, future research could focus on the association between family displacement due to job losses and cancer screening volume recovery. This could especially be of concern for those with low income, as being displaced may serve as an added barrier to receiving health care services. Third, although the Student–Newman–Keuls multiple-range test for multiple comparisons has several strengths, it does not adjust probability (*p*) values, which can potentially increase the risk of type 1 error when carrying out multiple statistical tests. Lastly, this analysis focuses on the impact of the COVID-19 pandemic on cancer screening volume recovery among a unique population of largely uninsured and low-income women aged 40–64 years for breast screening and aged 21–64 years for cervical screening. Therefore, the study results cannot be generalized to the total population of women in the US.

## 5. Conclusions

In conclusion, this study highlights the ongoing effects of the COVID-19 pandemic on screening volume in the NBCCEDP. Results show that NBCCEDP breast and cervical cancer screening volumes had not fully recovered to pre-COVID-19, 5-year averages by December 2022. Additionally, recovery in breast cancer screening volume was slower than that of cervical cancer, and racial/ethnic and geographic disparities in screening recovery were evident throughout the study period.

Screening volume recovery is essential, especially among lower-income populations that face greater challenges to healthcare. Study results can be used to inform strategies that can help reduce the risk of exacerbated cancer outcomes among a population of largely low-income and uninsured women served through the NBCCEDP. Specifically, strategies to improve cancer screening volume among racial/ethnic minority women may include increased efforts to identify and reduce race/ethnic specific barriers to screening and tailored outreach and support. On the other hand, strategies to improve screening among women in rural areas may include assisting patients with transportation barriers and helping to promote policies that address the broader issue of provider shortages.

While these strategies may be most ideal for helping to recover screening among women served by the NBCCEDP during the current 3-year study period, population differences in screening recovery will likely change over time. Therefore, we recommend future surveillance that examines population differences in the impact of the COVID-19 pandemic on cancer screening volume recovery three years after the COVID-19 pandemic was declared over (e.g., May 2023–May 2026). This would help to ensure that the most relevant strategies are being implemented and allow for trend comparisons, assessing screening volume before, during, and after the COVID-19 pandemic.

## Data Availability

The dataset analyzed for the current study comes from a restricted source—National Breast Cervical Cancer Early Detection Program data. This dataset can be made available by the corresponding author on reasonable request.
